# Exposure to socio-political unrest and wellbeing of older people in Hong Kong

**DOI:** 10.1186/s12877-022-03433-5

**Published:** 2022-09-23

**Authors:** Daniel W. L. Lai, Emma H. S. Liu, Elsie C. W. Yan, Jessica J. Li, Vincent W. P. Lee

**Affiliations:** 1 Faculty of Social Sciences, Hong Kong Baptist University, Room 1325, 13/F Academic and Administration Building, Baptist University Road Campus, Kowloon Tong, Hong Kong, China; 2 Department of Applied Social Sciences, The Hong Kong Polytechnic University, Hung How, Hong Kong, China; 3 Department of Social Work, The Chinese University of Hong Kong, Ma Liu Shui, Hong Kong, China; 4 Department of Social Work, Hong Kong Baptist University, Kowloon Tong, Hong Kong, China

**Keywords:** Social unrest, Mental health, Coping strategies, Older adults, Hong Kong

## Abstract

**Background:**

The social unrest in the second half of 2019 in Hong Kong came with conflicts, confrontations, and violence which affected almost everyone in the city. The destruction and disruption of the urban facilities have undoubtedly had a significant impact on the lives and mental well-being of the public, and the older people are even more vulnerable. This study examined the impacts of the social unrest on the wellbeing of older people, an area that was seldomly addressed in the public discourse and literature.

**Methods:**

Narrative interviews were conducted to capture older people’s lived experiences and ways of making sense of the unrest in Hong Kong. A total of 63 participants aged 60 and above was recruited through personal networks of the research team, and referrals by participants who took part in the interviews. Qualitative semi-structure interviews was conducted one on one via telephone.

**Results:**

Thirty-three male and 30 female participants took part in the interview. The number of participants from different risk zones affected by political unrest was comparable. Three themes were generated. Participants experienced challenges during the social unrest, including disturbance to family and social life, reduced incomes which affect quality of life, and difficulties in socializing with friends and accessing medical services. The social unrest caused emotional disturbance, giving rise to feelings of panic, fear, insomnia, depression, annoyance, and anger. Participants reported different coping strategies, ranging from moving to other places, to avoiding going to risky areas and watching news.

**Conclusion:**

Social unrest brings emotional distress to older people. In many cases, older people cope with challenges in different ways, whether active or passive. Social workers and other professionals should give more support to older people to encourage them to overcome their difficulties. The stakeholders’ awareness of the problem and mental health promotion is required to alleviate the multiple layers of negative impacts.

**Supplementary Information:**

The online version contains supplementary material available at 10.1186/s12877-022-03433-5.

## Background

In February 2019, the Government of the Special Administrative Region, the People’s Republic of China proposed to introduce the Fugitive Offenders and Mutual Legal Assistance in Criminal Matters Legislation (Amendment) Bill 2019 [1]. This bill has attracted fierce opposition from the public, giving rise to widespread protests, which were generally known as the anti-extradition law amendment bill movement. These protests have caused extensive damage to public facilities. Public transportation, government buildings, such as the Legislative Council building, and university campuses, such as the Chinese University of Hong Kong and the Hong Kong Polytechnic University, have been severely damaged [2, 3]. Over 3000 rounds of tear gas have been deployed as a means of crowd control by the police during the unrest [4]. Social unrest generally has a significant impact on the mental health of older adults. Experiencing political or social unrest could render older people vulnerable to negative emotions. Collective actions, such as protests, riots, and armed conflicts, are associated with depression, posttraumatic stress disorder (PTSD) and anxiety [5]. A study found that the Occupy Central Movement in 2014 brought negative effects on people’s mental health, as about 40% respondents reported self-perceived deterioration in emotions or sleeping quality compared to the pre-movement period [6].

In the context of this major socio-political unrest, the well-being of older adults appeared to be overlooked. Conflicts between younger and older generations have emerged in public areas and the media [7]. Higher prevalence of depression and anxiety is also reported among older people affected by political conflict compared with their younger counterparts [8]. Given their vulnerable conditions, older adults would suffer from additional worries and experience lower social and personal safety, which are associated with depression among older people in the Chinese context [9]. In short, older adults may be vulnerable to social unrest due to their limited health functions, physiological vulnerability, and greater reliance on social support and public services, particularly in times of turmoil. It is believed that older adults are especially vulnerable in terms of mental health during social unrest. A study suggested that the impact of social media was associated with the depression of older adults in social unrest [6]. Because of different political views, older adults may have heated debates with younger generation in both face-to-face and online interactions. Unlike the younger people, older adults’ network on social media were made up of family members, while it would be more upsetting for the older adults to have disagreement or debates with the loved ones. In other words, social media might be a factor which brought greater psychological distress to the older adults than the younger generations [6].

Moreover, a few studies suggested that the 2019 social unrest in Hong Kong was associated with psychological distress among older adults. It was found that older adults had a higher rate of probable depression than other age groups in 2019 social unrest [5]. Besides, older adults tended to be less likely to seek professional help when facing health problems related to social unrest [5]. Another study suggested that the political unrest in 2019 was correlated with suicidal ideation [10].

Given these challenges to older adults’ mental health, it is important to understand how older adults responded to the social unrest in Hong Kong and how the unrest impacted their mental health. Research on health and well-being has increasingly focused on empowerment and resilience, rather than pathological outcomes and weaknesses [11, 12]. This perspective is particularly important for this study because older people have generally been considered weak. This study adopts a strength-based approach to understand older people’s experiences in social unrest. This is based on the belief that people have the capacity to handle disruptions and challenges, including in conflict settings [13]. Effective coping helps people to develop resilience in everyday situations and in crisis [12]. This study aims to identify these ‘modifiable moderators’ between political violence and mental health. This study aimed to explore older people’s experiences of the socio-political unrest from four aspects using a bottom-up approach of qualitative study:

1. How were the experience of the older Chinese during the time of social unrest in Hong Kong?
2. What were the challenges they faced?
3. How did these experiences and challenges affect their mental health?
4. How did they cope with the challenges, particularly those related to their mental health?

## Method

Narrative interviews were used to explore people’s lived experiences and ways of making sense of the unrest. This method centres on participants’ own stories about their experiences from their perspectives [14]. Spontaneous storytelling can capture participants’ ways of managing their life under certain conditions [15]. The interview guide drew on the McGill Illness Narrative Interview Protocol as a systematic framework for understanding health experiences [16]. This study incorporated questions about experiences of unrest incidents and mental health in each section of the protocol (e.g., how people described mental health conditions, how they coped with mental health problems, how mental health and unrest affected their lives).

Through personal networks of the research team, and referrals by participants who took part in the interviews, a total of 63 participants completed the qualitative telephone interviews. Participants were recruited from communities with various frequencies of conflicts and damage. Some communities (e.g., Yau Tsim Mong, Yuen Long) were considered ‘high-risk’ zones with frequent conflict incidents, others (e.g. Shatin, Wong Tai Sin) as ‘medium-risk’, with fewer disruptions, and others (e.g. Southern District, Kowloon City) as ‘low-risk’. Around 20 participants were recruited from each zone, including men and women aged 60–75 and 75+, through local NGOs serving older adults, and personal networks using snowball sampling to reach those who were not affiliated with service providers. Each interview lasted about 45 minutes to 1 hour. Data collection was done by trained research assistants. A semi-structured interview guide was used to guide the interview process. The guide covers topics related to the impact of the social unrest, disruptions experienced, coping strategies, interpretation and perspectives towards the social unrest, and views on expectations of future development. The interview guide is included as the supplementary file. This research received ethics approval from the Human Subjects Ethics Sub-Committee of The Hong Kong Polytechnic University (HSEARS20191205001–02) and the research was carried out in accordance with the relevant guidelines and regulations, in line with the Helsinki Declaration. Verbal informed consent was obtained from all the interviewees prior to the interview as the interview was conducted over telephone. The way in which the study was conducted and the use of verbal informed consent were approved by the ethics committee.

Data analysis was conducted using the MAXQDA software. All the interviews were audiotaped and transcribed verbatim, and the verbatim were checked against the tapes for accuracy. Inductive content analysis was used to identify themes from the interview. Initial codes were generated through reading and coding the whole dataset and then all the codes were sorted into potential themes. The differences of the codes and themes were analysed based on their significance to the research questions. The conformability of the trustworthiness could be ensure as the coding process was independently carried out by two researchers. The simultaneous analysis by two researchers minimises any potential bias or personal motivation on the part of the researcher and ensures that the content presented is the true meaning of the participant. Candidate themes and subthemes were selected and reviewed among the research team to ensure that these themes were internally coherent, accurate and distinctive.

### Background of participants of qualitative telephone interviews

A total of sixty-three participants took part in the qualitative interviews. The demographics of the participants are shown in Table 1.

**Table 1:** Demographics of participants in qualitative interviews (*n* = 63)

	Number (%)
**Age group**	
60–64	3 (4.8%)
65–69	29 (46%)
70–74	28 (44.4%)
75–80	3 (4.8%)
**Gender**	
Male	33 (52.4%)
Female	30 (47.6%)
**Risk Zone**^**a**^	
High-risk	19 (20.2%)
Medium-risk	20 (31.7%)
Low-risk	24 (38.1%)

^a^High-risk zone: Yau Tsim Mong, Yuen Long, Sham Shui Po, Central & Western, Wan Chai

Medium-risk zone: Shatin, Wong Tai Sin, Tuen Mun, Eastern District, Tai Po, Sai Kung, Tsuen Wan

Low-risk zone: Southern District, Kowloon City, Kwun Tong, Island, Northern District, Kwai Tsing

## Results

**Fig. 1:**
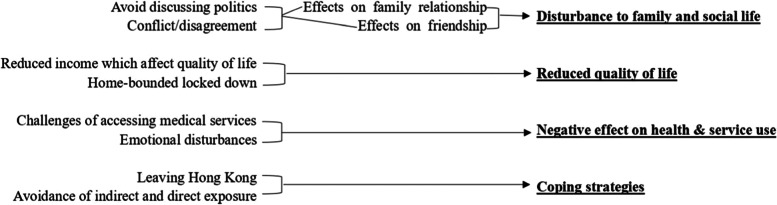
Coding tree for qualitative analysis of qualitative telephone interview

Three themes including: the experiences of older Chinese people and challenges confronting them during social unrest in Hong Kong, emotional disturbances and coping strategies, were generated from the qualitative data analysis. A coding tree (Fig. 1) was produced so as to provide a clear structure and overall picture of the meaning of the whole dataset.

### The experiences of older Chinese people and challenges confronting them during social unrest in Hong Kong

#### Disturbance to family and social life

Participants reported experiencing changes in their relationships with their family members. Some of them had conflicts with their family members, especially younger ones, because of different opinions towards this incident. Some of them intentionally avoided talking about the incident with their family members to avoid conflict. For example, Participant 31 (male, 68 years old with tertiary education, residing in a high-risk zone) said,

“The incident affects my everyday life. It separates my family members… For example, I said I think there is no problem if the protest is legal…Things like that. Then we start to have different opinions.”

Moreover, some participants also experienced conflicts with their friends because of different opinions towards this incident. Some of them intentionally avoided talking about the incident with their friends to avoid conflicts. Some even quitted WhatsApp groups due to different political views from their friends. For example, Participant 20 (male, 80 years old with vocational education, residing in a medium-risk zone) reported,

"I had quarrel with my friends…Some of them disagree with the young people, because they hate them destroying the facilities and the society. I say the ones who destroy the facilities may not be the young people who are at school. Then they said they are sure that they are students. After that conversation, we are not happy.”

#### Reduced income which affects quality of life

A number of participants reported the loss of or decline in income. Some participants reported encountering difficulties in taking public transportation and reaching destinations they preferred, which affected their work and participation in activities. Social unrest posed a huge challenge to their finances, which further affected them psychologically. Participant 38 (female, 66 years old with secondary education, residing in medium-risk zone) talked about the effect of the loss of income on three of his children. He said,

“For example, if there is movement, then I could not be able to go to that district, because there is no traffic… My son couldn’t be able to back to work. Then he had no income. It lasted for around two months. So helpless… It also made them have psychological problems, made them unhappy.”

#### Home-bounded lockdown

Many participants thought that the home-bounded lockdown posed a disturbance to their daily life and mental health. For example, Participant 44 (female, 73 years old, residing in a low-risk zone) reported feeling afraid of going to public places, while she used to socialize with her friends in public before the pandemic. She said,

“We dare not go anywhere. Do you think it’s a good thing? Living in here, I don’t know what to do…I am afraid of going to malls… I even need to speak quietly…”

#### Challenges of accessing medical services due to closure of public transportation

Apart from the psychological and physiological impact, some participants with existing illnesses experienced inconvenience in going to hospitals for further consultation with doctors. They reported difficulties in accessing medical services due to damages to pubic facilitates and disruption to public transportation, such as the MTR. Participant six (female, 73 years old with tertiary education, residing in low-risk zone), who had difficulties in walking due to her feet problem, said,

“I have to see doctor regularly…Now I see the doctor twice a week. When the incident was very intense, the MTR sometimes closed. It affects me a lot… At the beginning, I took taxi, but it’s too expensive and not cost-effective. It costs me more than $100 (Hong Kong Dollar) each time. But I really cannot walk by myself, so there is no way.”

### Emotional disturbances

Many participants revealed that social unrest caused emotional disturbance to them. It affected their daily life in the following ways.

#### Feelings of panic and fear

Feelings of panic and fear were the most common emotions shared by participants. Some of the participants witnessed the incident in person and some watched news on TV. Participant two (female, 74 years old with secondary education, residing in high-risk zone), who witnessed the incident in person, said,

“When I was shopping, I was so scared when I bumped into a huge crowd of people… My heart trembles, my feet tremble...I didn't know where to go... I was so scared. I felt much better after I left that area... I dare not go out even when I need to go outside and buy food or meet people… Well, it affected my daily life and my social activities.”

#### Feeling angry and annoyed

Some participants expressed that their daily lives were seriously affected by the incident. They felt annoyed by the interruption regardless of their political stands. Some participants also reported feeling angry due to the violent behaviours of the police and the behaviours of the protestors. Participant three (male,74 years old with tertiary education, residing in a high-risk zone) who lived in Yau Tsim Mong District, shared how her everyday life was affected in detail,

“If I took an 8-minute walk to the Nathan Road in Mong Kok, it was a mess. I heard ambulance and police sirens during the day and at night... You ask me how I feel, it's helpless.”

#### Feeling depressed

Several participants felt depressed because they witnessed severe damage to the city. Some other participants reported feeling depressed due to the government’s attitude towards the protestors. For example, Participant 19 (male, 72 years old residing in a low-risk zone) said:

“A lot of things have been destroyed. It takes a lot of money and resources to fix it...like the universities were destroyed like that. So crazy! It’s really heart-breaking. Alas... (Bitter laugh)”

#### Having difficulties in falling asleep/insomnia

Participants also mentioned that thinking about the series of socio-political incidents affected their nerves and led to different levels of insomnia. Most participants who reported suffering from insomnia had witnessed violent and traumatic events during this socio-political incident. For example, participant seven shared her experience of having difficulties in falling asleep.

“One of my most memorable incidents was seeing a policeman step on young man's head with one foot, break his arm, and step on his foot... After seeing these negative things, I couldn't sleep until 6 o'clock the next morning…This symptom lasted for about one and half month.”

### Coping strategies

#### Leaving Hong Kong

When asked about their coping strategies, some participants reported having thought of or preparing to leave Hong Kong and migrate to other places, given the negative impact of the socio-political incident on their lives. Some of them felt hopeless at the future of Hong Kong. For example, Participant one (male, 68 years old residing in a medium-risk zone) said,

“We didn’t participant in the movement. We will not participant in it…If something bad happens, we can leave Hong Kong. We can live somewhere else anyways. We might move back to Mainland China or overseas…”

#### Avoidance of indirect and direct exposure

Many participants motioned trying to avoid watching news about the incident to reduce mental discomfort. They, therefore, avoided or spent less time watching news. They tried to distract themselves by engaging in other activities, such as meeting friends and attending activities. For example, Participant 34 (female, 69 years old with secondary education residing in a medium-risk zone) reported,

“My anxiety is the anxiety of seeing these images of the incident…I used to watch TV all the time. Now I watch less…If I feel too emotional when watching TV, I will change the channel to relax myself.”

For participants who were living in risky areas with frequent conflicts reported, some of them avoided going out. For participants living in lower risk zones, what they did was to avoid going to higher risk zones. For example, Participant 60 (female of 69 years old with secondary education, residing in low-risk zone), who was living in Aberdeen, said,

“In the past, I used to go out by myself. Now I find the society so dangerous, so I don’t dare go out by myself…I find nowhere is safe. When I see strangers, I avoid them. I stay in Aberdeen and within the community.”

## Discussion

This study represents one of the first studies to provide new empirical evidence of the impact of social unrest on older people’s daily life and their ways of coping. There has only been limited study of the psychological impact of socio-political movements [17, 18], not to mention the impact on older people. The findings of this study show that conflicts and disruptions arising from the social unrest have affected the daily routines of older people, including their family and social life and access to medical services, and given rise to negative emotions. Meanwhile, the coping strategies reported by participants show that older people should not be generalized as ‘weak’ or ‘powerless’. Instead, they are, to a greater or lesser degree, able to handle disruptions and challenges. Although the strategies adopted were mainly using passive or avoidance approaches, their capacity and ability to face the difficult time should not be undermined. The discovery serves to support the fact that even in difficult and challenging situations, the strength of individual aging adults is able to be adopted as the source of reliance. However, due to the fact that negative coping strategies may be perceived as being negative, service providers should consider promoting alternative options for the older people in future. These findings have important implications for healthcare service and policy in their efforts to further prevent challenges and difficulties faced by the older people in the event of other social unrest situations at the societal level.

Most participants revealed that social unrest caused emotional disturbance to them. This echo previous studies showing that older people are vulnerable to depression during social unrest [9, 19]. In addition, not only did older people experience challenges to access public services, but they also encountered family conflicts in the private sphere. The findings show that older people faced arguments and quarrels with younger people and that the intergenerational relationships were affected. These results could be understood within the Hong Kong context where local people have become more sensitive to political issues and outspoken about their political views [18]. Before Hong Kong’s handover to mainland China, it is commonly believed that Hong Kong people tended to be indifferent to politics and put more emphasis on maintaining harmonious family relations and enhancing family well-being [20]. A recent survey showed that the prevalence of depression among Hong Kong people increased during the social unrest (‘Depression Numbers Now at a 10-Year High’, 2019). In other words, the trends of political polarizations and confrontations in Hong Kong play a more important role in Hong Kong people’s daily lives nowadays [18]. Echoing these results, the interview data show that people’s different views towards politics and consequent family conflicts may have a negative impact on older people’s relationships with their younger family members and their mental health. More public education and government measures are needed to enhance intergenerational relationships and communication within families.

On the other hand, the study echoes the existing literature that individuals are equipped with the capacity to cope with difficulties, including in conflict settings [13]. Although some participants might adopt a more passive approach to avoiding conflicts by avoiding discussions with their younger family members, some also expressed that they actively engaged in other activities, such as meeting friends and attending activities in order to avoid watching news about the unrest. These findings show that older people should not be generalized as ‘weak’ or ‘powerless’. Instead, they are able to handle disruptions and challenges. Previous research has suggested that one of the important ways for enhancing well-being during the social unrest is to savor daily positive events and emotions [18, 21]. Bryant and Veroff [21] suggested three key strategies for savouring, including reminiscing past positive experiences, savouring the moment, and anticipating upcoming positive experiences. Building on these insights and the findings, older people should be encouraged to engage in social activities and savour positive emotions from these activities.

The findings have important implications for public health. Different stakeholders in society, namely government officials, mental health professionals, and the media, should be sensitive to the vulnerability of older people to the threats of social unrest. Research has suggested that effective coping depends on one’s social resources, which enable or restrict one’s ability to meet personal needs in everyday life and in conflict settings [12]. In other words, older people, particularly those seriously troubled by the social unrest, should be given more support to develop positive coping strategies to handle emerging socio-political stressors. World Health Organization [22] calls for more action to launch mental health promotion programs because these programs can facilitate people’s adjustment to socio-political changes and adverse social environment. Social workers and other professionals can play a role in supporting older people to develop effective coping strategies. For instance, they should encourage older adults to share their feelings and emotions about the news or incidents happening around them in order to help them relieve their emotions. Alternatively, social workers can sum up the news and information about the social incidents with their clients, and set up their own dissemination platforms for older adults. They can also consider using cognitive behavioural therapy to change the pessimistic attitudes or negative emotions among older people. By doing so, they can create a supportive and inclusive environment to guide older people to overcome their emotions in the face of social unrest.

Several limitations of the study warrant attention. Given a relatively small sample of Hong Kong Chinese older people, the findings cannot be seen as generalizable to the older adult population or other communities in Hong Kong. Also, it is possible that people with serious mental issues or those who were not concerned about political issues were less likely to participate in this study. Telephone interview might also be one of the limitations due to its shortcoming of being unable to capture participants’ body language and eye contacts etc. Having said that, this study potentially unfolds a relatively new research direction towards the impact of social unrest on older people as uncertainties in socio-political development are commonly observed worldwide. Similar research should be conducted among subgroups, such as those with high and low socio-economic status and different religions because mental health promotion programs would require different strategies for different communities.

## Conclusion

To conclude, the findings show that many older adults were negatively affected by the social unrest in Hong Kong. They faced disturbance to their daily, family, and social lives, and encountered difficulties in accessing medical services. While some older people were able to develop coping strategies, different stakeholders’ awareness of the problem and mental health promotion are needed to alleviate the negative impact.

## Supplementary Information

**Additional file 1.** Qualitative personal interview guide.

## Data Availability

Supporting data and data analysis materials are available from the corresponding author (Prof. Daniel, Lai) upon request.
