# Supplementary material for: Exposure to socio-political unrest and wellbeing of older people in Hong Kong
Source: BMC Geriatr. 2022 Sep 23;22:768. doi: 10.1186/s12877-022-03433-5 (PMC9502941; doi:10.1186/s12877-022-03433-5)
Supplement: Supplementary file 1 — Additional file 1. Qualitative personal interview guide. [file 12877_2022_3433_MOESM1_ESM.docx]

Qualitative personal interview guide

1. Demographic background: age, gender, residential district, educational background, living arrangement, employment (current occupation or occupation before retirement), socio-economic status
2. What do you think about the recent social and political incident? How would you describe the incident?
3. In what aspect do you think the recent social and political incident has affected your daily routine?
4. In what ways have you changed your daily routine recently because of the incident (coping strategies)?
5. How would you describe your current mental health and wellbeing? What are the differences before and after the social and political incident in terms of your mental health and wellbeing?
6. What are the challenges you face in terms of your mental health and wellbeing?
7. How do you respond to the negative influences of social and political incident on your mental health and wellbeing?
8. What are the main factors that affect your everyday life and your mental health and wellbeing?
9. How would you describe your relationship with your family members? Does it change before and during the incident? Are there any conflicts/debates concerning the current incident within your family?
10. How would you describe your interpersonal relationship? Does it change before and during the incident?
11. What do you think about the future development of the recent social and political incident?
12. What are your expectations towards the roles of different sectors in the recent social and political incident? (e.g. the government, the police, students, or the younger generation?)
